# Supplementary material for: Gut dysbiosis narrative in psoriasis: matched-pair approach identifies only subtle shifts correlated with elevated fecal calprotectin
Source: Microbiol Spectr. 2024 Dec 10;13(1):e01382-24. doi: 10.1128/spectrum.01382-24 (PMC11705824; doi:10.1128/spectrum.01382-24)
Supplement: Supplemental figures — Fig. S1 to S5. [file spectrum.01382-24-s0001.pdf]

SUPPLEMENTARY MATERIAL:

Gut dysbiosis narrative in psoriasis: matched-pair approach identifies only subtle shifts correlated with elevated fecal calprotectin

Bayazit Yunusbayev,[1,2]# Anna Bogdanova,[3] Nadezhda Nadyrchenko,[4] Lavrentii Danilov,[2] Viktor Bogdanov,[5,6] Grigory Sergeev,[3] Radick Altinbaev,[7] Fanil Bilalov,[8,9] Milyausha Yunusbaeva,[1,2]

[1]Institute of Translational Biomedicine, Saint-Petersburg State University, Saint-Petersburg, Russia

[2]Department of Genetics and Biotechnology, Saint-Petersburg State University, Saint-Petersburg, Russia

[3]ITMO University, Saint-Petersburg, Russia

[4]Republican Dermatovenerologic Dispensary, Ufa, Russia

[5]Moscow Institute of Physics and Technology, Moscow, Russia

[6]Federal Research Center for Innovator and Emerging Biomedical and Pharmaceutical Technologies, Moscow, Russia

[7]Institute of Higher Nervous Activity and Neurophysiology of RAS, Moscow, Russia

[8]Bashkir State Medical University, Ufa, Russia

[9]Republic Medical Genetic Centre, Ufa, Russia

Supplementary Figures

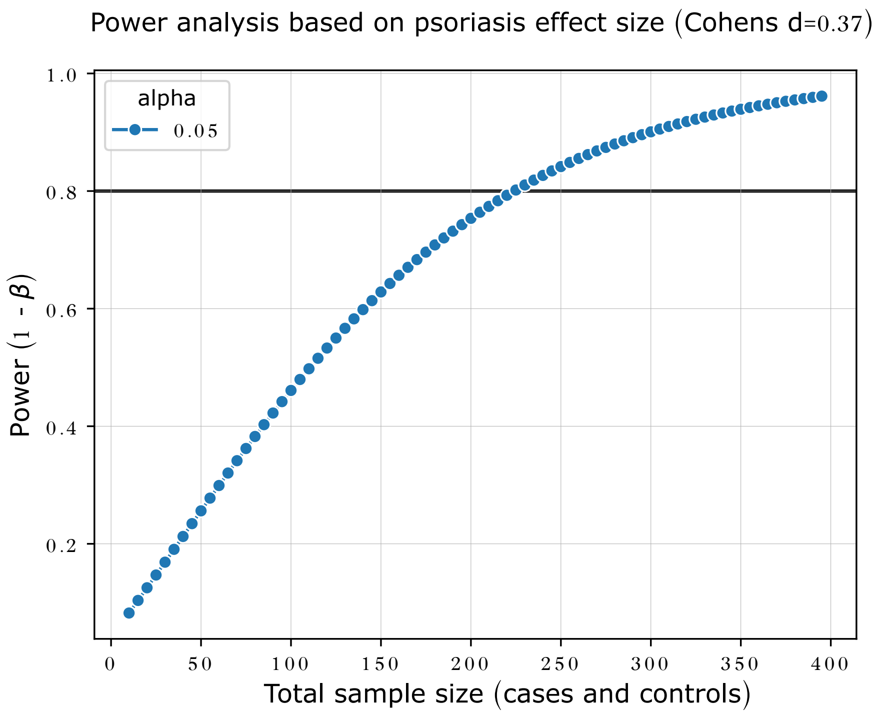

Figure S1 Power analysis based on estimated effect size for psoriasis

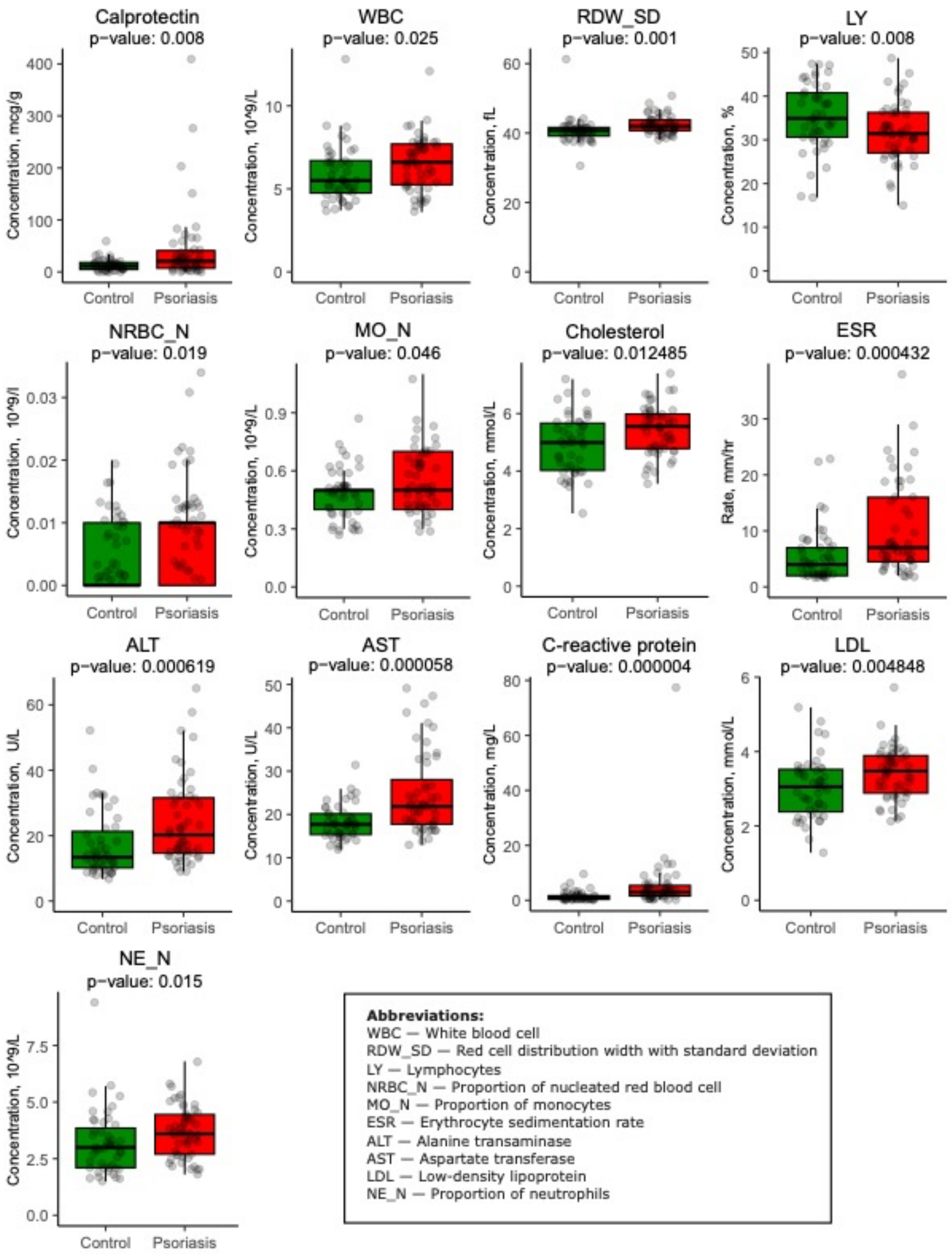

Figure S2 Host biomarkers with statistically significant ($P \leq 0.05$) differences (14 out of 38 measured) between patients and healthy controls.

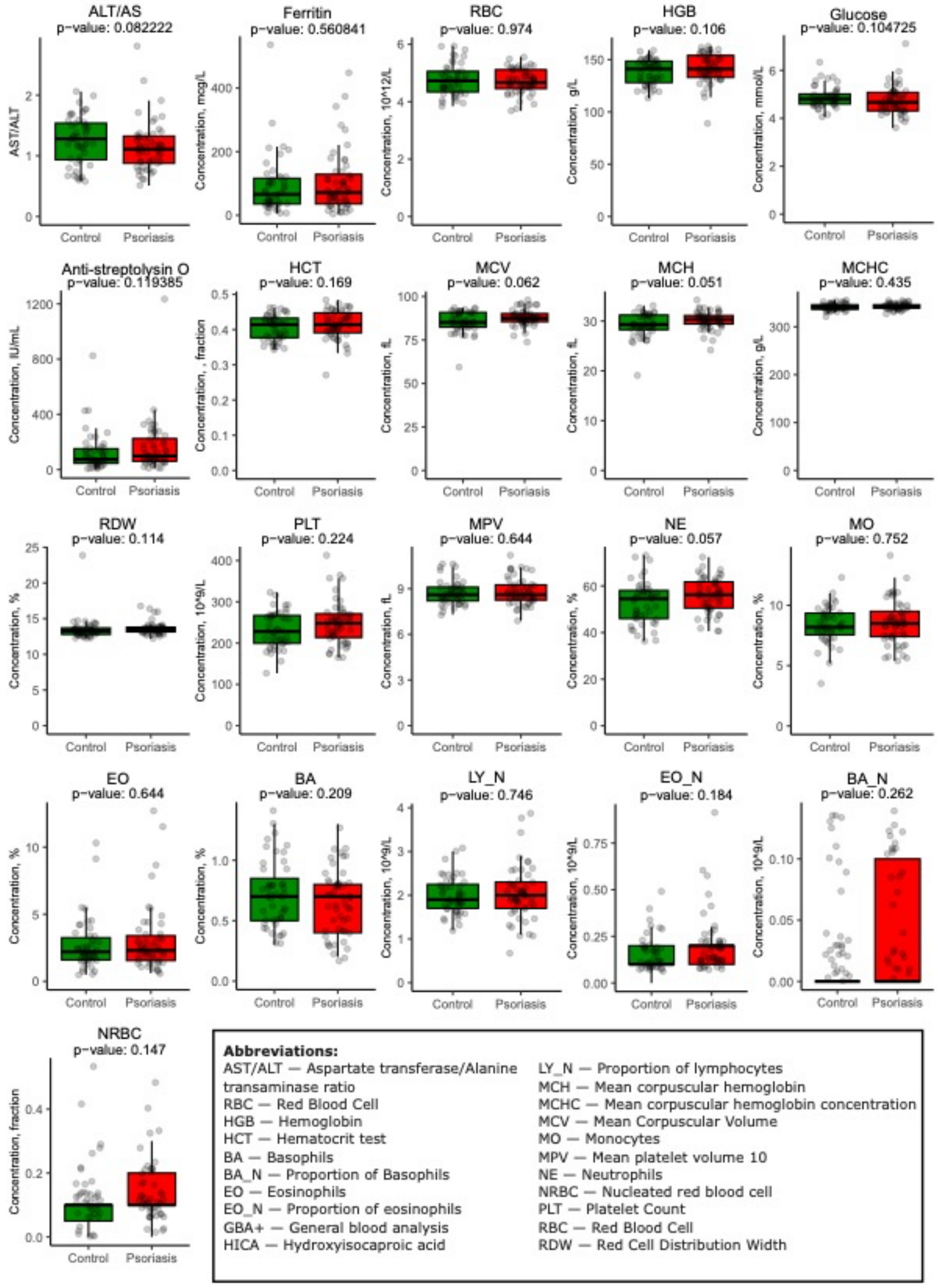

Figure S3 Host biomarkers with no significant ($P > 0.05$) differences (21 out of 38 measured) between patients and healthy controls.

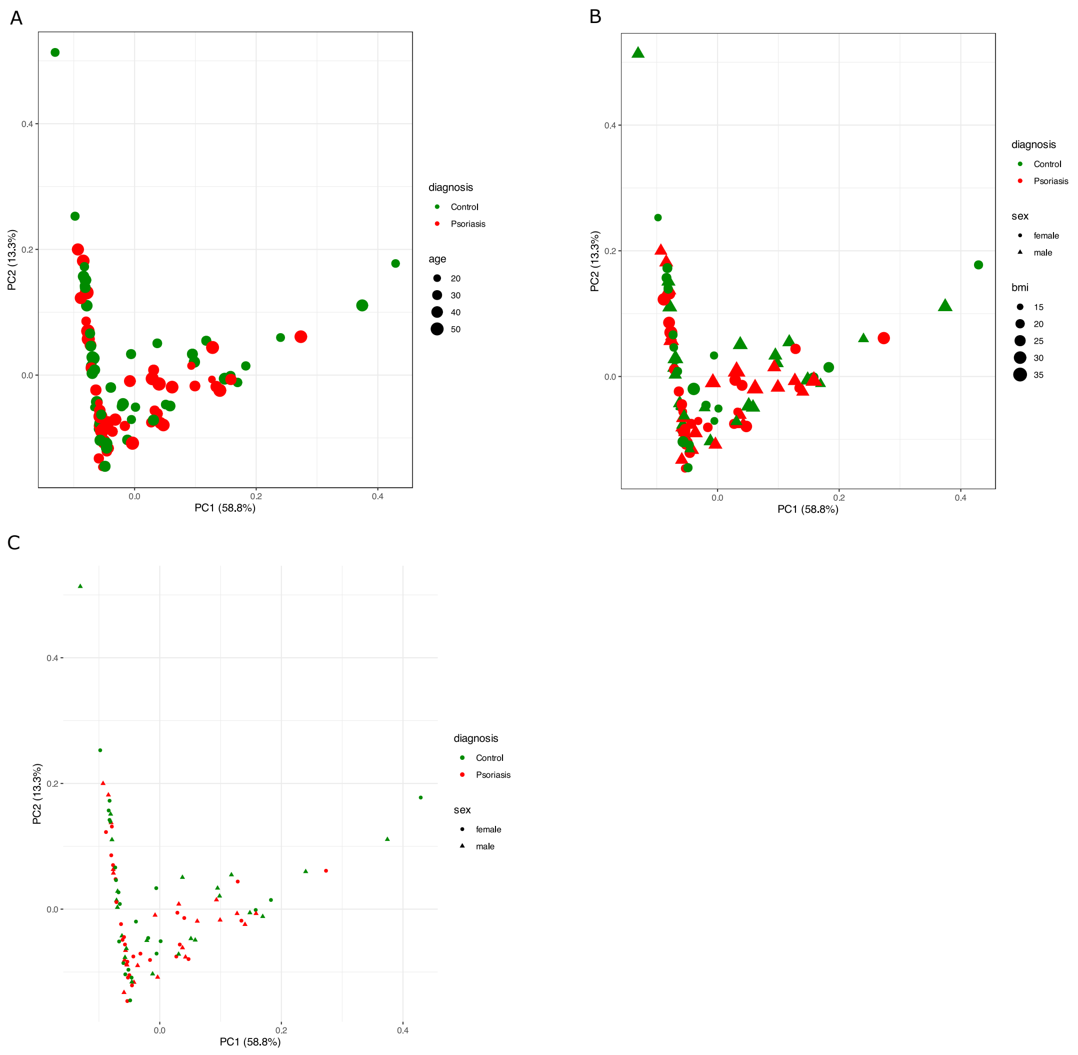

Figure S4 Distribution of age, sex, and body mass index (BMI) among patients and controls projected on principal components of microbiome variation.

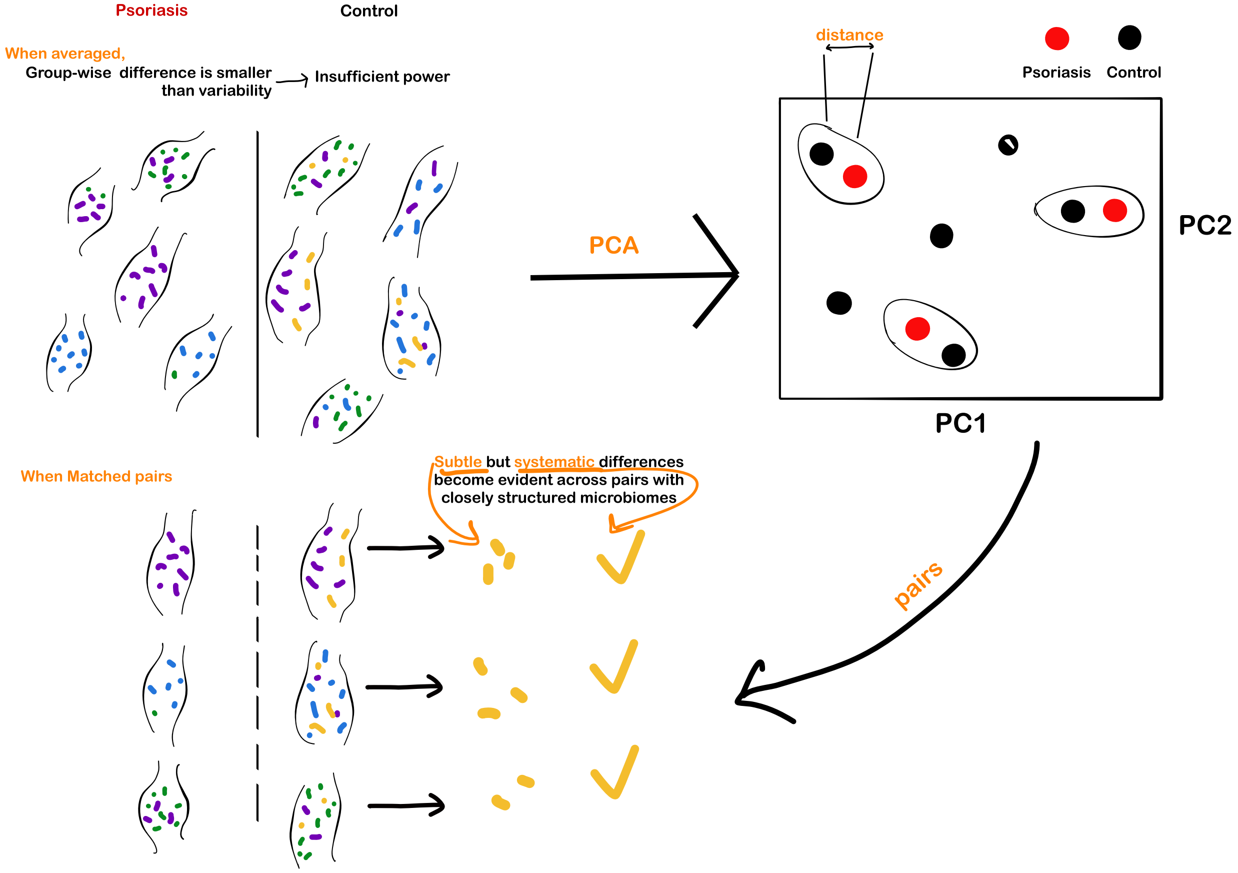

Figure S5 Schematic diagram representing our matched-pair approach

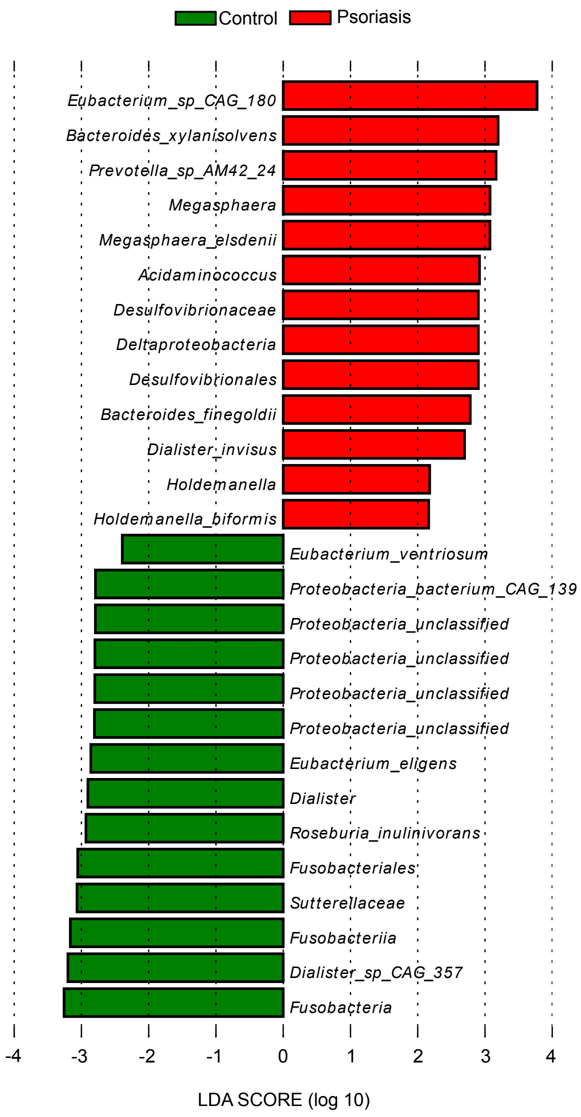

Figure S6 Differentially abundant bacterial species inferred using the linear discriminant analysis, LEfSe.
