# Supplementary material for: Gut dysbiosis narrative in psoriasis: matched-pair approach identifies only subtle shifts correlated with elevated fecal calprotectin
Source: Microbiol Spectr. 2024 Dec 10;13(1):e01382-24. doi: 10.1128/spectrum.01382-24 (PMC11705824; doi:10.1128/spectrum.01382-24)
Supplement: Table S1 — Short summaries of 12 published case-control gut microbiome studies. [file spectrum.01382-24-s0002.docx]

**Table S1** Short summaries of 12 published case-control gut microbiome studies

| Study | Dataset (N) | Origin | Method | Criteria (N, taxonomic resolution) | Alpha diversity | Ordination | Multivariate comparison | Differential abundance test | Differentially abundant taxa | Reference |
| --- | --- | --- | --- | --- | --- | --- | --- | --- | --- | --- |
| Tan et al. 2018 | Psoriasis (14), Controls (14) | China, Changsha, Patients ethnic origin is not indicated | 16S rRNA sequencing | N<30 | No difference in alpha diversity (Shannon, p>0.05) | Patients and controls overlap and were not clearly separated on the PCA plot | NA | Non-parametric t-test using Metastats software | a) At the phylum level, there is a decreased proportion of Verrucomicrobia and Tenericutes in psoriasis patients. b) At the class and order level, there was a reduced proportion of Mollicutes and Verrucomicrobiae, Verrucomicrobiaes, and RF39 in psoriasis patients. c) At the family level, there was a decreased proportion of Verrucomicrobiaceae and S24-7 , but an increased proportion of Bacteroidaceae and Enterococcaceae in patients. d) At the genus level, Akkermansia was less abundant, while the abundance of Enterococcus and Bacteroides was increased in patients with psoriasis. | https://onlinelibrary.wiley.com/doi/10.1111/exd.13463 |
| Codoñer FM et al.2018 | Psoriasis (52), Controls (300) | Spain, Alicante, Patients ethnic origin is not indicated | 16S rRNA sequencing | N>30 and HC>30, Low taxonomic resolution | Diversity higher in patients (Shannon,p < 0.001) | Patients and controls overlap and were not clearly separated on the PCA plot | NA | DESeq2 approach to identify differentially abundant counts | a) At the genus level, there is an increased proportion of Faecalibacterium, Akkermansia and Ruminococcus. Decreased proportion of Bacteroides in patients. | https://www.nature.com/articles/s41598-018-22125-y |
| Hidalgo-Cantabrana et al.2019 | Psoriasis (19), Controls (20) | Spain, Astirias, Patients ethnic origin is not indicated | 16S rRNA sequencing | N<30 | Diversity lower in patients (Shannon, p‐value<0.014) | Patients and controls separated on three‐dimensional PCoA plot | Clustering of patients and controls (PERMANOVA, p‐value<0.001) | ANCOM statistical framework for compositional data | a) At phylum level, reduced proportion of Bacteroides and Pseudomonadota (Proteobacteria), but increased proportions of Actinobacteria and Firmicutes in patients. b) At genus level, Blautia, Bifidobacterium, Collinsella and Slackia were significantly overrepresented in the patients, whereas Bacteroides, Parabacteroides, Barnesiella, Alistipes and Paraprevotella were significantly underrepresented. | https://academic.oup.com/bjd/article/181/6/1287/6752122 |
| Shapiro et al. 2019 | Psoriasis (24), Controls (22) | Israel, TelAviv, Patients ethnic origin is not indicated | 16S rRNA sequencing | N<30 | No difference in alpha diversity (Shannon, p>0.05) | Patients and controls separated on two‐dimensional PCoA plot | NA | Linear Discriminant Analysis (LefSe) | a) At phylum level, reduced proportion of Bacteroidetes and Pseudomonadota (Proteobacteria), but increased proportion of Firmicutes and Actinobacteria in patients. b) At genus level, increased proportion of Blautia, Faecalibacterium, Ruminococcus, Coprococcus, Bifidobacterium, and Dorea, but decrease in the Prevotella in patients. At the species level, there were significant increases in the relative proportions of Ruminoccocus gnavus, Dorea formicigenerans and Collinsella aerofaciens in patients, while Prevotella copri was significantly increased in the control group. | https://onlinelibrary.wiley.com/doi/10.1111/1346-8138.14933 |
| Dei-Cas I. et al. 2020 | Psoriasis (55), Controls (27) | Argentina, Patients ethnic origin is not indicated | 16S rRNA sequencing | N>30 but HC<30, Low taxonomic resolution | NA | Patients and controls largely overlap on the PCA plot | NA | Linear Discriminant Analysis (LefSe) | a) At phylum level, reduced proportion of Bacteroidetes, but increased proportion of Firmicutes in patients. b) At genus level, Faecalibacterium and Blautia (both belong to the phylum Firmicutes, class Clostridia and order Clostridiales) had higher proportion in patients, and Bacteroides and Paraprevotella were more abundant in controls. | https://www.nature.com/articles/s41598-020-69537-3 |
| Yegorov S. et al.2020 | Psoriasis (14), Control (7) | Kazakhstan, Nursultan, Patients ethnic origin is not indicated | 16S rRNA sequencing | N<30 | No difference in alpha diversity (Shannon, p>0.05) | PCoA mentioned in Methods section but results not presented | ANOVA mentioned in Methods section but results not presented | Linear Discriminant Analysis (LefSe) | a) At phylum level, Firmucutes/Bacteroides ratio had tendency to be elevated but difference was not statistically significant. b) At the family level, reduced proportion of Lachnospiraceae, but increased abundance of Ruminococcaceae. c) At genus level, Faecalibacterium was elevated in patients, whereas Oscillibacter and Roseburia had higher proportion in controls. IL1-a was significantly increased in fecal samples from patients compared to controls. | https://www.frontiersin.org/articles/10.3389/fimmu.2020.571319/full |
| Xiao S. et al. 2021 | Psoriasis (30), Control (15) | China, Beijing, Chinese | Shotgun sequencing | N=30 but HC<30, Low taxonomic resolution | No difference in alpha diversity (Shannon, p>0.05) | Patients and controls largely overlap on the PCoA plot | PERMANOVA mentioned in Methods section but results not presented | Linear Discriminant Analysis (LefSe) | a) At phylum level, Firmicutes, Actinobacteria and Verrucomicrobia Roseburia had tendency to be increased, but Bacteroidetes, Euryarchaeota and Pseudomonadota (Proteobacteria) were reduced in proportion in patients; b) At family level, decreased proportion of the Oxalobacteraceae, Porphyromonadaceae, Pasteurellaceae, Rikenellaceae, Sphingobacteriaceae and Comamonadaceae in patients. c) At the genera level, increased proportion of Faecalibacterium, Bacteroides, Bifidobacterium, Megamonas and Roseburia and a decreased proportion of Butyricimonas, Oxalobacter, Actinobacillus, Odoribacter, Anaerotruncus, Alistipes, Pseudoflavonifractor, Pedobacter, and Comamonas in patients. Alterations in predicted functional potential: a) 15 KEGG pathways were predicted to be enriched in patients microbiomes (including LPS biosynthesis, WNT signaling pathway, apoptosis, bacterial secretion system, phosphotransferase system, and others) b) 8 KEGG pathways were were predicted to be depleted patients (bacterial secretion system, ribosome, fructose and mannose metabolism, galactose metabolism, phosphotransferase system, LPS biosynthesis, valine, leucine and isoleucine biosynthesis, glycine, serine and threonine synthesis). c) 5 metabolites were predicted to be dysregulated in a subset of patients microbiomes (H2S, isovalerate, isobutyrate, hyaluronan and hemicellulose). | https://www.frontiersin.org/articles/10.3389/fcimb.2021.605825/full |
| Chang H. W. et al. 2022 | Psoriasis (33), Control (15) | USA, San Francisco, Patients ethnic origin is not indicated | Shotgun sequencing | N>30 but HC<30 | No difference in alpha diversity (Shannon, p>0.05) | Patients and controls largely overlap on the PCoA plot | NA | Negative binomial model after controlling (DEseq2) | a) At species level, top 7 species with positive log2 fold-change in patients: Prevotella_disiens, Clostridium_sp_KLE_1755, Bacteroides_coprocola, Erysipelotrichaceae_bacterium_5_2_54FAA, Dorea_unclassified, Prevotella_buccae, Brachyspira_unclassified*,* and top 7 negative log2 fold-change: Campylobacter hominis, Ruminococcus champanellensis, Corynebacterium glutamicum, Bacteroides sp_2_1_22, Butyricicoccus pullicaecorum, Citrobacter unclassified, Bacteroides sp_1_1_6. | https://www.sciencedirect.com/science/article/pii/S2667026722000236#fig1 |
| Schade L. et al. 2022 | Psoriasis (21),  Control (24) | Brazil, Curitiba, Patients ethnic origin is not indicated | 16S rRNA sequencing | N<30 | NA | Patients and controls largely overlap on the PCA plot | NA | QIIME 2 bioinformatics platform (Exact method used for differential abundance analysis was not mentioned) | a) At genus and species level, increased proportion of Dialister and Catenilbacterium genera and the Prevotella copri; Decreased proportion of Ruminococcus, Lachnospira and Blautia genera, as well as the Akkermansia muciniphila. | https://academic.oup.com/lambio/article-abstract/74/4/498/6989204?redirectedFrom=fulltext&login=false |
| Todberg et al. 2022 | Psoriasis (53),  Control (52) | Denmark, Patients ethnic origin is not indicated | Shotgun sequencing | N>30 and HC>30, Comparable taxonomic resolution | No difference in alpha diversity (Shannon, p>0.05) | Patients and controls largely overlap on the PCoA plot. However, authors report significant differences between communities (p=0.01) | Three clusters were delineated and patients were enriched in cluster 3 (p=0.023) | Dirichlet multinomial mixtures approach | a) At phylum level, Actinobacteria and Euryarchaeota were increased in those with more severe disease. b) At family level, Methanobacteriaceae was increased with more severe disease. Alterations in predicted functional potential: Increased methanogenesis and decreased butyrate production were associated with higher PASI, although these were borderline significant (FDR‐corrected P = 0·13 and 0·13). | https://doi.org/10.1111/bjd.21245 |
| Wen C. et al. 2023 | Psoriasis (32), Control (32).  17 Control samples: spouses of psoriasis patients | China, Beijing, Han Chinese | Shotgun sequencing | N>30 and HC>30, Comparable taxonomic resolution | No difference in alpha diversity (Shannon, p>0.05) | Patients and controls largely overlap on the PCoA plot | PERMANOVA, Centroids and dispersion of the groups are equivalent (p=0.279) | Linear Discriminant Analysis (LefSe) | a) At phylum level, reduced proportion of Firmicutes, but increased proportion of Bacteroidetes in patients* b) At genus level, Roseburia and Eubacterium proportions were lower in patients. c) At species level,  increased proportion of Bacteroides uniformis, and Escherichia unclissified, but decreased proportion of Roseburia hominis.  2) Three KEGG pathways were predicted to be enriched in patients microbiomes (lipopolysaccharide biosynthesis  (ko00540), ribosome (ko03010) and central carbon metabolism in cancer (ko05230); Four KEGG pathways were predicted to be depleted (flagellar assembly (ko02040), ABC transporters (ko02010), Starch and sucrose metabolism (ko00500) and bacterial chemotaxis (ko02030).  **Psoriasis (17) vs Healthy spouses (17)**  1) Species: ↑ Alistipes finegoldii, Alistipes onderdonkii; ↓ Bacteroides eggerthii  2) KEGG: ↑ ribosome (ko03010) and peptidoglycan biosynthesis (ko00550); ↓ bacterial chemotaxis (ko02030) and the phosphotransferase system (PTS) (ko02060) | https://www.sciencedirect.com/science/article/pii/S0882401023000037?via%3Dihub |
| Xiao Y et al., 2024 | Psoriasis (44),  Psoriatic arthritis (26),  Control (25) | China, Sichuan,  Patients ethnic origin is not indicated | Shotgun sequencing | N>30 but HC<30, Low taxonomic resolution | No difference in alpha diversity (Shannon, p>0.05 | Patients and controls overlap and were not clearly separated on the PCA plot | PERMANOVA mentioned in Methods section but results not presented | Linear Discriminant Analysis (LefSe) | a) Eubacterium rectale, Alistipes finegoldii and A. shahii were highly depleted in psoriasis patients, but Eubacterium hallii, Streptococcus thermophilus, Bifidobacterium animalis, and Megasphaera unclassified were increased.  b) 4 KEGG pathways (starch and sucrose metabolism, bacterial chemotaxis, flagellar assembly, butanoate metabolism) were significantly depleted in psoriasis patients compared to that in control | <https://journals.asm.org/doi/10.1128/spectrum.01154-23> |

*The only study out of 12 that shows diminished Firmicutes but increased Bacteriodes in patients
