# Supplementary material for: Gut dysbiosis narrative in psoriasis: matched-pair approach identifies only subtle shifts correlated with elevated fecal calprotectin
Source: Microbiol Spectr. 2024 Dec 10;13(1):e01382-24. doi: 10.1128/spectrum.01382-24 (PMC11705824; doi:10.1128/spectrum.01382-24)
Supplement: Table S2 — Top 14 bacterial species with abundance shifts in patients based on matched-pair Wilcoxon test. [file spectrum.01382-24-s0003.docx]

**Table S2** Top 14 bacterial species with abundance shifts in patients based on matched-pair Wilcoxon test

| Bacterial species | P-value | FDR | Enrichment rank | Occurence (Controls) | Occurence (Patients) | Rank (Control) | Rank (Patients) |
| --- | --- | --- | --- | --- | --- | --- | --- |
| Megasphaera_elsdenii | 0.0018 | 0.438 | psoriasis | 0.1277 | 0.4043 | 40.89 | 54.11 |
| Eubacterium_sp_CAG_180 | 0.009 | 0.5757 | psoriasis | 0.2128 | 0.5106 | 39.84 | 55.16 |
| Rothia_mucilaginosa | 0.0129 | 0.5757 | psoriasis | 0.0851 | 0.1702 | 45.2 | 49.8 |
| Ruminococcus_obeum_CAG_39 | 0.0144 | 0.5757 | control | 0.1915 | 0.1064 | 49.94 | 45.06 |
| Bacteroides_xylanisolvens | 0.0176 | 0.5757 | psoriasis | 0.766 | 0.8936 | 40.82 | 54.18 |
| Parasutterella_excrementihominis | 0.0184 | 0.5757 | control | 0.8298 | 0.7447 | 52.83 | 42.17 |
| Haemophilus_sp_HMSC71H05 | 0.0195 | 0.5757 | control | 0.5106 | 0.2979 | 53.31 | 41.69 |
| Clostridium_leptum | 0.0219 | 0.5757 | psoriasis | 0.2979 | 0.4468 | 42.83 | 52.17 |
| Dialister_sp_CAG_357 | 0.0225 | 0.5757 | control | 0.1489 | 0.0213 | 50.51 | 44.49 |
| Catenibacterium_mitsuokai | 0.0231 | 0.5757 | psoriasis | 0.234 | 0.3617 | 43.89 | 51.11 |
| Eubacterium_eligens | 0.0297 | 0.6722 | control | 0.8936 | 0.6809 | 55.82 | 39.18 |
| Prevotella_sp_AM42_24 | 0.036 | 0.7477 | psoriasis | 0.0213 | 0.1277 | 44.96 | 50.04 |
| Roseburia_inulinivorans | 0.0454 | 0.8085 | control | 1,0000 | 0.8936 | 55.23 | 39.77 |
| Bacteroides_salyersiae | 0.0455 | 0.8085 | control | 0.2128 | 0.1064 | 50.21 | 44.79 |

Species in red have increased proportion in patients
