# Supplementary material for: Gut dysbiosis narrative in psoriasis: matched-pair approach identifies only subtle shifts correlated with elevated fecal calprotectin
Source: Microbiol Spectr. 2024 Dec 10;13(1):e01382-24. doi: 10.1128/spectrum.01382-24 (PMC11705824; doi:10.1128/spectrum.01382-24)
Supplement: Table S3 — Top bacterial species associated with a linear increase in calprotectin. [file spectrum.01382-24-s0004.docx]

**Table S3** Top bacterial species associated with a linear increase in calprotectin

| Bacterial species | RDA-score | PC1 | PC2 | PC3 | PC4 | PC5 |
| --- | --- | --- | --- | --- | --- | --- |
| **Eubacterium_sp_CAG_180** | **0.0797** | **0.0606** | **-0.0224** | **-0.0276** | **0.0195** | **-0.0279** |
| **Megasphaera_elsdenii** | **0.0786** | **0.0516** | **-0.0321** | **-0.0247** | **0.0114** | **0.0516** |
| **Catenibacterium_mitsuokai** | **0.0742** | **0.0877** | **-0.0605** | **-0.0186** | **-0.0196** | **-0.0151** |
| **Phascolarctobacterium_succinatutens** | **0.0566** | **0.064** | **-0.0339** | **-0.019** | **-0.0127** | **-0.0557** |
| **Prevotella_copri** | **0.0527** | **0.1363** | **-0.0628** | **-0.0943** | **-0.0031** | **-0.0471** |
| **Desulfovibrionaceae_bacterium** | **0.0481** | **0.0515** | **0.0309** | **0.0032** | **-0.0021** | **0.0106** |
| **Bacteroides_sp_CAG_530** | **0.046** | **0.0394** | **-0.0265** | **-0.0129** | **-0.0083** | **-0.0259** |
| Clostridium_symbiosum | 0.0448 | -0.0511 | -0.0003 | -0.0242 | -0.0283 | -0.01 |
| Clostridium_bolteae | 0.0444 | -0.1233 | -0.0114 | -0.0762 | -0.0054 | -0.0585 |
| Clostridium_asparagiforme | 0.041 | -0.0585 | 0.0331 | -0.0037 | 0.0101 | -0.0096 |
| Ruminococcus_sp_CAG_330 | 0.0402 | 0.0202 | -0.0224 | -0.0005 | -0.008 | -0.0029 |
| Sutterella_parvirubra | 0.04 | 0.0538 | 0.003 | -0.0348 | 0.0604 | -0.0205 |
| Slackia_isoflavoniconvertens | 0.0394 | 0.0923 | -0.0069 | -0.0051 | 0.0236 | -0.049 |
| Firmicutes_bacterium_CAG_170 | 0.039 | 0.0903 | -0.0073 | 0.046 | -0.0833 | -0.0061 |
| Allisonella_histaminiformans | 0.0368 | 0.0383 | -0.0006 | -0.0084 | 0.0463 | 0.0297 |
| Bacteroides_clarus | 0.0348 | 0.0134 | 0.0844 | 0.003 | 0.0088 | -0.0166 |
| Acidaminococcus_fermentans | 0.0334 | 0.0245 | -0.0187 | -0.0019 | 0.001 | -0.0092 |
| Paraprevotella_xylaniphila | 0.0321 | 0.0495 | 0.0584 | -0.064 | -0.011 | 0.0357 |
| Harryflintia_acetispora | 0.0308 | -0.0121 | 0.0286 | -0.0198 | -0.0082 | -0.0124 |

Altogether, 249 species were tested with Redundancy analysis
